# A systematic review and meta-analysis of the relationship between advanced glycation end products ceceptor (RAGE) gene polymorphisms and the risk of inflammatory bowel disease

**DOI:** 10.22088/cjim.14.3.41

**Published:** 2023

**Authors:** Ratih Puspita Febrinasari, Sagitaisna Putri Indah, Eka Rezkita Bastomy, Steven Irving, Akhmad Azmiardi, Rabbinu Rangga Pribadi, Marcellus Simadibrata, Yulia Sari

**Affiliations:** 1 Department of Pharmacology, Faculty of Medicine, Universitas Sebelas Maret, Surakarta, Indonesia; 2 Faculty of Medicine, Universitas Sebelas Maret, Surakarta, Indonesia; 3 School of Health Sciences Mamba'ul 'Ulum, Surakarta, Indonesia; 4 Division of Gastroenterology, Pancreatobiliary and Digestive Endoscopy, Department of Internal Medicine, Faculty of Medicine, Universitas Indonesia, Cipto Mangunkusumo National Central General Hospital, Jakarta, Indonesia; 5 Department of Parasitology, Faculty of Medicine, Universitas Sebelas Maret, Indonesia

**Keywords:** Inflammatory bowel disease, Advanced glycation end products receptor, Genetic polymorphisms, Meta-analysis, Systematic review.

## Abstract

**Background:**

In the pathogenesis of inflammatory bowel disease (IBD), the advanced glycation end product receptor (RAGE) has been involved. IBD is classified into Chron’s disease (CD) and ulcerative colitis (UC). The promoter gene of the RAGE gene was discovered to have had unique polymorphisms that increased its transcriptional activity. This study, therefore, used a systematic review and meta-analysis to examine the relationship between the RAGE gene polymorphism and the risk of IBD.

**Methods:**

Databases such as PubMed, Scopus, and Cochrane library were searched to identify the relationship between RAGE gene polymorphisms and IBD susceptibility. We identified three Single Nucleotide Polymorphism (SNPs) (RAGE-429T/C, 374T/A, and G82S). The data were analyzed by RevMan 5.4.

**Results:**

Four studies (932 cases/1366 controls) were included. The findings showed no relationship between RAGE –429T/C and –G82S polymorphisms and the risk of IBD in all genetic models significantly. TT genotype of RAGE –374T/A polymorphisms was related to increased CD risk (OR=1.37; 95%CI=1.04-1.81; P=0.02), while TA genotype was determined to be a protective factor (OR=0.75; 95%CI=0.57-0.99; P=0.04). In UC, A allele of RAGE -374T/A was related to increase risk (OR=1.26; 95%CI=1.04-1.53; P=0.02), while T allele was determined to decrease risk (OR=0.79; 95%CI= 0.65-0.96; P=0.02).

**Conclusions:**

Our findings demonstrated that TT genotype and A allele of RAGE -374T/A polymorphisms were related to CD and UC risks, respectively, while the TA genotype and T allele possibly had a protective effect. RAGE –429T/C and RAGE –G82S polymorphisms were not related to increased IBD risk.

IBDs are the condition of chronic inflammation of the gastrointestinal system, including Chron's disease (CD) and ulcerative colitis (UC). There were around 6 to 8 million IBD cases worldwide in 2017 (1). Both ulcerative colitis and Chron’s disease affect men and women equally. They might occur in adolescents and adults (2, 3). Some CD and UC symptoms comprise diarrhea, rectal bleeding, stomach ache, and loss of weight. IBD is shown by recurrent episodes of gastrointestinal tract inflammation brought on by an aberrant immune reaction to gut bacteria (3). IBD is caused by a number of pathogenic causes, including aberrant gut microbiota, environmental changes, dysregulated immune responses, and genetic variations. IBD occurs often in family aggregation, which suggests that genetic factors have a crucial involvement in the development of IBD (4). 

The capacity of the RAGE receptor to attach to advanced glycation end-products, whose particular polymorphisms of the promoter gene were identified to boost its transcriptional activity, is one of its main functions (5). RAGE gene is found in the so-called class III of the major histocompatibility complex, on the short arm of chromosome 6: 6p21.3 and take a role in immune responses (6). RAGE is highly expressed during embryonic development, but generally found at low levels in the most tissues of healthy adults such as in intestinal epithelium (7). These days, RAGE is known as a receptor that is associated with some pathophysiological diseases. One of them is IBD (7-9). The expression and functionality of the RAGE gene are impacted by functional polymorphisms. It could make people more vulnerable to IBD (5, 7-11). 

The regulatory components of the RAGE gene contain a number of single nucleotide polymorphisms (SNPs), such as G82S, -374T/A, -492T/C, and others.^5 ^The putative binding location of the receptor experiences an amino acid exchange as a result of the G82S RAGE polymorphism. The -374T/A RAGE promoter polymorphism increases transcriptional activity via decreasing the attachment of a nuclear factor to a regulatory region of the RAGE gene promoter. However, data on the -429T/C promoter polymorphism are scarcer. Recently, studies have found that RAGE polymorphisms were associated with IBD, but the results are inconclusive. Research carried out by Wang et al. showed that the RAGE G28S variant genotype was associated with UC susceptibility (10). Study by Ciccociopo et al. demonstrated that TT genotype of -374 T/A polymorphism was related to early onset of CD. In contrast, the AA genotype was related to the late onset of CD (8). These genetic variants may impact the RAGE expression or function and are related to IBD (7, 12). RAGE polymorphisms may play a role in IBD; however, it is not yet apparent. Hence, we investigated the relationship between the RAGE gene polymorphism and the risk of IBD by analyzing the three most important RAGE polymorphisms (G82S, -374T/A, and -429T/C). 

## Methods

**Strategy of search:** To find pertinent studies from the time of publication to July 1, 2022, systematic literature searches of electronic databases comprising PubMed, Scopus, and the Cochrane library were carried out, utilizing the following terms: “Inflammatory Bowel Disease,” “IBD” “Chron disease,” “Ulcerative Colitis,” “advanced glycation end products receptor,” “RAGE,” “Genetic polymorphisms,” “SNP”. The language was restricted in English. The study was expanded to incorporate the bibliographies of every study that was qualified. A manual search of reviews of lifespan research was also conducted to find any more studies that could be pertinent. Then, authors were contacted personally if needed for any more information needed.

**Selection of study: Inclusion and exclusion criteria: **The articles for the meta-analysis were chosen based on the inclusion criteria listed below: (i) case-control; (ii) cohort; (iii) comparative study; (iv) case group was a group of patients diagnosed with either CD or UC according to clinical, radiological, endoscopic, and pathology criteria; (v) control group was a group of healthy individual having neither a personal nor family history of IBD, nor a history of malignancy; (vi) the primary outcome was the relationship between the RAGE G82S, -374T/A, and -429T/C gene polymorphism and the risk of IBD; (vii) supplied enough genotype information to compute the odds ratio (OR) and 95% confidence range (CI). Meanwhile, here is the list of exclusion criteria: if any of the following conditions apply: i) there was no control group; ii) the distribution genotypes in the placebo group did not match with Hardy-Weinberg equilibrium (WHE); iii) the study populations overlapped; iv) there were small numbers more than 100 cases; v) the articles were not available in English; vi) the findings were only reported in conference proceeding book.

**Extraction of data: **We were adhered to The Meta-analyses of Observational Studies in Epidemiology (MOOSE) guidelines. The online supplement contains the comprehensive strategies of search. Two authors (B.R. and I.S.P.) independently assessed the titles and abstracts of publications after receiving preliminary database search results to weed out studies that did not answer the main study issue. Each included study's reference list was thoroughly investigated to find other pertinent researches. The remaining publications' entire texts were then separately evaluated by the same two authors (B.R. and I.S.P.) to determine if they offered pertinent and comprehensive material using the predetermined inclusion and exclusion criteria, as described below. Additionally, the bibliographies of the chosen publications and review articles on the subject were looked into to find any undiscovered research.

 Then, the reviewers' discussion helped to settle any disagreements amongst the authors about the inclusion of studies (A.A., R.P.F., B.R., I.S.P, RRP, MS and S.I). In addition, we used information from the most recent thorough report, where duplicate studies from the same cohort were found. Author/s, year of publication, ethnicity, number of sample (cases and controls), and genotype/allele frequencies were all retrieved. If not expressly supplied, information on HWE was also manually estimated or retrieved.

**Assessment of study quality: **Included studies were assessed using the Newcastle-Ottawa Scale (NOS), which assigns a point from 0 (the lowest) to 9 (maximum). There are eight elements on this scale, divided into three groups (selection, comparability, and outcome). Studies were rated as high quality if they had a total score of six or above, moderate quality if they had a score of four to six, and low quality if they scored lower or equal to four (13). 

**Statistical analysis: **For polymorphisms examined in at least four studies, meta-analysis was done. A Review Manager (Rev-Man) 5.4 was used to analyze the data. The relationship between the gene polymorphisms and longevity were estimated using allelic ratios and 95% confidence intervals. The inverse variance approach was applied to a random or fixed-effects model. We used Z test to assess the significance of the pooled OR. Study heterogeneity was characterized as low (25%), medium (50%) or high (75%), and the I^2^ statistic was used to determine the amount of variance across the results owing to study heterogeneity rather than sampling error. No significant heterogeneity was stated by an I^2^ score of less than 50% and/or a p-value of 0.05. Forest plots were prepared for each study. After sequentially removing each included study, sensitivity analyses were carried out to determine how each one affected the aggregate OR. Visual examination of funnel plots was used to assess potential publication bias.

**Critical appraisal: **A critical review was carried out using the Oxford Center for Evidence-Based Medicine (CEBM) which included the validity, importance, and applicability of the articles that had been selected (14). 

## Results

**Figure 1:**
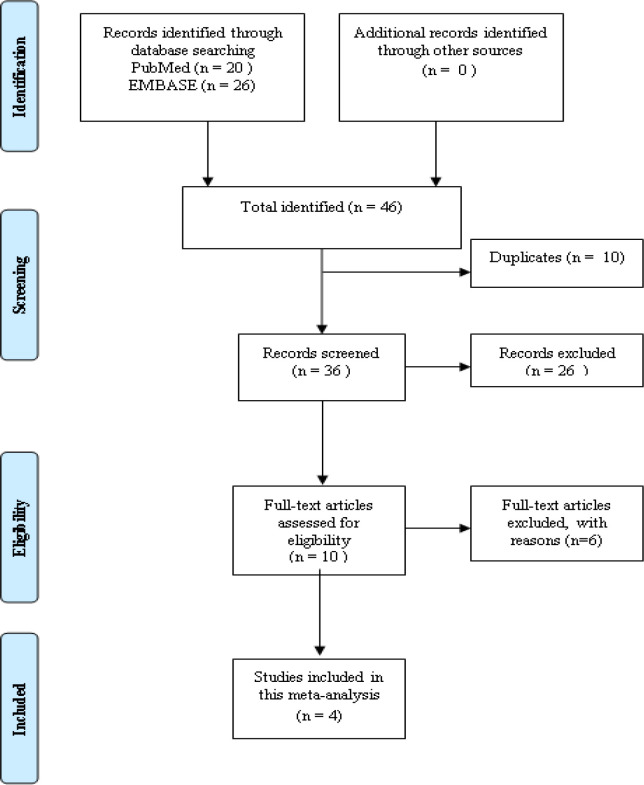
Flowchart of selection process for meta-analysis

**Included Studies’ Characteristics:** Figure 1 showed the study selection flow diagram. After eliminating five duplicate data, a total of 46 articles were found using publication search. By reviewing the titles and abstracts of 26 papers, we eliminated them since they had nothing to do with RAGE SNPs.

 In our meta-analysis, ten studies were then analyzed. Six of them lacked original data, and we were unable to get in touch with their writers. Our meta-analysis, which comprised four papers with total of 932 cases and 1336 controls, was included. Table 4, 5, 6 cover the eligible studies’ characteristics and Newcastle-Ottawa Scale (NOS) ratings.

**Table 1:** Baseline characteristics of studies regarding RAGE -429T/C gene polymorphisms and inflammatory bowel disease

**Study**	**Year**	**Country**	**Ethnic**	**Type of IBD**	**Sample Size (Case/Control)**	**Case**							**Control**							**Genotyping**	**X** ^2^ ** HWE**
						**TT**	**TC**	**CC**	**N**	**T**	**C**	**n**	**TT**	**TC**	**CC**	**N**	**T**	**C**	**n**		
Ciccocioppo et al.	2019	Italy	Caucasian	CD	133/161	74	47	12	133	195	71	266	118	41	2	161	277	45	322	PCR-RFLP	0.5628
Ciccocioppo et al.	2019	Italy	Caucasian	UC	149/161	112	33	4	149	257	41	298	118	41	2	161	277	45	322	PCR-RFLP	0.5628
Wang et al.	2014	China	Asian	CD	312/479	228	82	2	312	538	86	624	353	118	8	479	824	134	958	PCR-LDR	0.2713
Wang et al.	2015	China	Asian	UC	72/479	52	20	0	72	124	20	144	353	118	8	479	824	134	958	PCR-LDR	0.2713
Wang et al.	2016	China	Asian	UC	266/247	179	81	6	266	439	93	532	185	58	4	247	428	66	494	HRM	0.0505

**Critical appraisal: **We extracted four studies and evaluated the evidence level of these included articles utilizing the CEBM criteria. The assessment results show that these four articles have level 2B. Level 2B includes individual prospective cohort studies. Level 2 is considered to have enough evidence that is sufficiently strong and consistent. 

**Involved RAGE SNPs’ Characteristics: **The meta-analysis included three RAGE SNPs in total: RAGE-429T/C, 374T/A, and G82S. Tables 1,2,3 display their basic data, function predictions, and genotype frequency distributions for all these SNPs. Basic data is divided into ethnic (including Caucasian and Asian) and IBD types. Tables 1, 2, 3 show that the most samples are from Asian ethnicity and the majority of IBD sufferers with UC types. Numerous records were left out of the quantitative synthesis because they did not follow HWE (PHWE 0:05) or because there were insufficient data for some loci. 

**Table 2:** Baseline characteristics of studies regarding RAGE -374T/A gene polymorphisms and inflammatory bowel disease

**Study**	**Year**	**Country**	**Ethnic**	**Type of IBD**	**Sample Size (Case/Control)**	**Case**							**Control**							**Genotyping**	**X** ^2^ ** HWE**
						**TT**	**TA**	**AA**	**N**	**T**	**A**	**n**	**TT**	**TA**	**AA**	**N**	**T**	**A**	**n**		
Ciccocioppo et al	2019	Italy	Caucasian	CD	133/161	52	55	26	133	159	107	266	59	75	27	161	193	129	322	PCR-RFLP	0.1449
Ciccocioppo et al.	2019	Italy	Caucasian	UC	149/161	41	69	39	149	151	147	298	59	75	27	161	193	129	322	PCR-RFLP	0.1449
Wang et al.	2014	China	Asian	CD	312/479	248	62	2	312	558	66	624	343	123	12	478	809	147	956	PCR-LDR	0.0602
Wang et al.	2015	China	Asian	UC	72/479	46	22	4	72	114	30	144	343	123	12	478	809	147	956	PCR-LDR	0.0602
Wang et al.	2016	China	Asian	UC	266/247	157	106	4	267	420	124	544	146	93	8	247	385	108	493	HRM	2.2184

**Table 3:** Baseline characteristics of studies regarding RAGE -G82S gene polymorphisms and inflammatory bowel disease

**Study**	**Year**	**Country**	**Ethnic**	**Type of IBD**	**Sample Size (Case/Control)**	**Case**							**Control**							**Genotyping**	**X** ^2^ ** HWE**
						**GG**	**GS**	**SS**	**N**	**G**	**S**	**n**	**GG**	**GS**	**SS**	**N**	**G**	**S**	**n**		
Wang et al.	2014	China	Asian	CD	312/479	174	112	26	312	460	164	624	303	148	28	479	754	204	958	PCR-LDR	2.9309
Wang et al.	2015	China	Asian	UC	72/479	48	22	2	72	118	26	144	303	148	28	479	754	204	958	PCR-LDR	2.9309
Wang et al.	2016	China	Asian	UC	266/247	134	123	9	266	391	141	532	179	60	8	247	418	76	494	HRM	1.1083

**Table 4:** Quality assessment of included studies of RAGE -429T/C gene polymorphisms and inflammatory bowel disease using the New-Castle Ottawa Scale (NOS)

**Study**	**Year**	**Selection**				**Comparability**	**Exposure**			**Total**
		**Case definition**	**Representativeness of the cases**	**Selection of controls**	**Definition of controls**		**Ascertainment of Exposure**	**Same method of ascertainment for all subjects**	**Non-Response Rate**	
Ciccocioppo et al.	2019	*	*	-	*	*	*	*	*	7
Wang et al.	2014	*	*	-	*	*	*	*	*	7
Wang et al.	2015	*	*	-	*	**	*	*	*	8
Wang et al.	2016	*	*	-	*	*	*	*	*	7

(*) means one point

**Figure 2:**
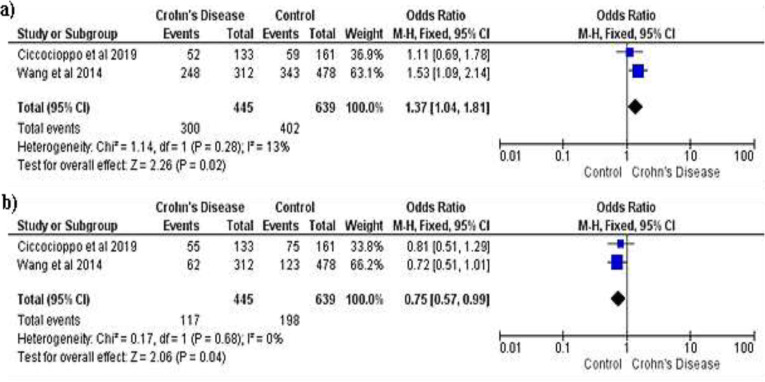
Forest plot of association between RAGE -374T/A gene polymorphisms and Crohn's Disease. a) TT vs TA+AA; b) TA vs TT+AA

**Figure 3:**
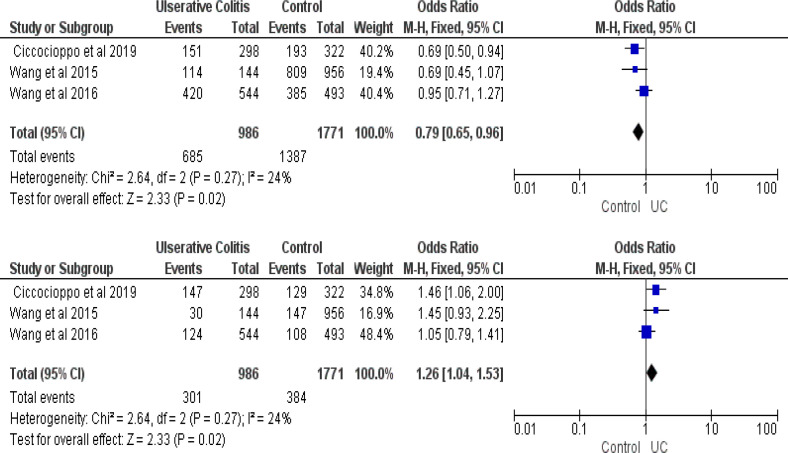
Forest plot of association between RAGE -374T/A gene polymorphisms and Ulserative Colitis. a) T vs A; b) A vs T

**RAGE SNPs and Inflammatory Bowel Disease Susceptibility: A Quantitative Data Synthesis: **First, the pooled ORs method was used to evaluate the relationship between RAGE SNPs and IBD susceptibility. There was no connection between the RAGE -429T/C polymorphism and the RAGE -G82S allele and IBD susceptibility in all genetic models (tables 7 and 9). Table 8 showed that The RAGE - 374T/A polymorphisms were discovered to be related to IBD risk. TT genotype of RAGE –374T/A polymorphisms were associated with increased CD risk (OR=1.37; 95%CI=1.04-1.81; P= 0.02), while TA genotype was related to decreased risk (OR=0.75; 95%CI=0.57-0.99; P= 0.04), with forest plot that showed on figures 2 and 3. In UC, A allele of RAGE -374T/A was related to increased risk (OR=1.26; 95%CI=1.04-1.53; P= 0.02), while T allele served as its protective factor (OR= 0.79; 95%CI=0.65-0.96; P= 0.02).

**Table 5:** Quality assessment of included studies of RAGE -374T/A gene polymorphisms and inflammatory bowel disease using the New-Castle Ottawa Scale (NOS)

**Study**	**Year**	**Selection**				**Comparability**	**Exposure**			**Total**
		**Case definition**	**Representativeness of the cases**	**Selection of controls**	**Definition of controls**		**Ascertainment of Exposure**	**Same method of ascertainment for all subjects**	**Non-Response Rate**	
Ciccocioppo et al.	2019	*	*	-	*	*	*	*	*	7
Wang et al.	2014	*	*	-	*	*	*	*	*	7
Wang et al.	2015	*	*	-	*	**	*	*	*	8
Wang et al.	2016	*	*	-	*	*	*	*	*	7

(*) means one point

**Table 6:** Quality assessment of included studies of RAGE -G82S gene polymorphisms and inflammatory bowel disease using the New-Castle Ottawa Scale (NOS)

**Study**	**Year**	**Selection**				**Comparability**	**Exposure**			**Total**
		**Case definition**	**Representativeness of the cases**	**Selection of controls**	**Definition of controls**		**Ascertainment of Exposure**	**Same method of ascertainment for all subjects**	**Non-Response Rate**	
Wang et al.	2014	*	*	-	*	*	*	*	*	7
Wang et al.	2015	*	*	-	*	**	*	*	*	8
Wang et al.	2016	*	*	-	*	*	*	*	*	7

(*) means one point

**Table 7:** The summary of the association between RAGE -429T/C gene polymorphisms and the risk of Inflammatory Bowel Disease

**Type of IBS**	**Allele & genotype**	**NS**	**Model**	**Value**		**Sensitivity (%)**	**Specifity (%)**	**OR**	**95%CI**	**p**_h_	**p**
				**Case (%)**	**Control (%)**						
CD	T vs C	2	Random	82.35%	86.01%	82.35%	13.98%	0.68	0.30 – 1.53	0.001	0.35
	C vs T	2	Random	33.33%	13.98%	33.33%	86.01%	1.46	0.65 – 3.28	0.001	0.35
	TT vs TC + CC	2	Random	67.86%	73.59%	67.86%	26.40%	0.68	0.33 – 1.42	0.01	0.31
	TC vs TT + CC	2	Fixed	17.59%	14.44%	17.59%	75.15%	1.22	0.93 – 1.61	0.21	0.15
	CC vs TT + TC	2	Random	8.91%	5.58%	8.91%	98.43%	1.74	0.09 – 34.1	0.006	0.72
UC	T vs C	3	Fixed	84.18%	86.18%	84.18%	13.81%	0.86	0.68 – 1.09	0.40	0.22
	C vs T	3	Fixed	23.47%	13.81%	23.47%	86.18%	1.16	0.92 – 1.48	0.40	0.22
	TT vs TC + CC	3	Fixed	70.43%	73.95%	70.43%	26.04%	0.84	0.64 – 1.10	0.33	0.21
	TC vs TT + CC	3	Fixed	16.34%	14.19%	16.34%	75.53%	1.18	0.90 – 1.54	0.27	0.24
	CC vs TT + TC	3	Fixed	6.49%	5.71%	6.49%	98.42%	1.30	0.53 – 3.22	0.59	0.54

Notes : NS = Number of samples, OR = odds ratio, CI = confidence interval, p = p value based on a between-study Z test, ph = p value based on Q test for heterogeneity between studies

**Figure 4:**
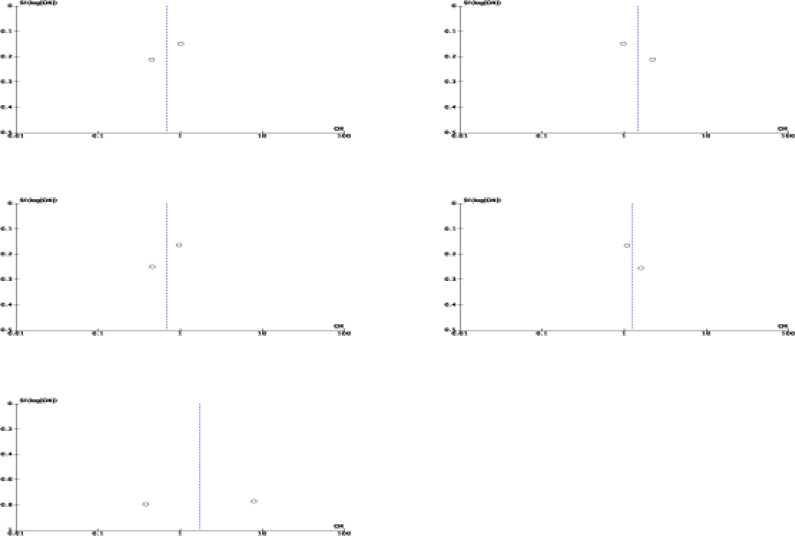
Funnel plot of association between RAGE -429T/C gene polymorphisms and Crohn's Disease. a) T vs C; b) C vs T; c) TT vs TC+CC; d) TC vs TT+CC; e) CC vs TT+TC

**Figure 5:**
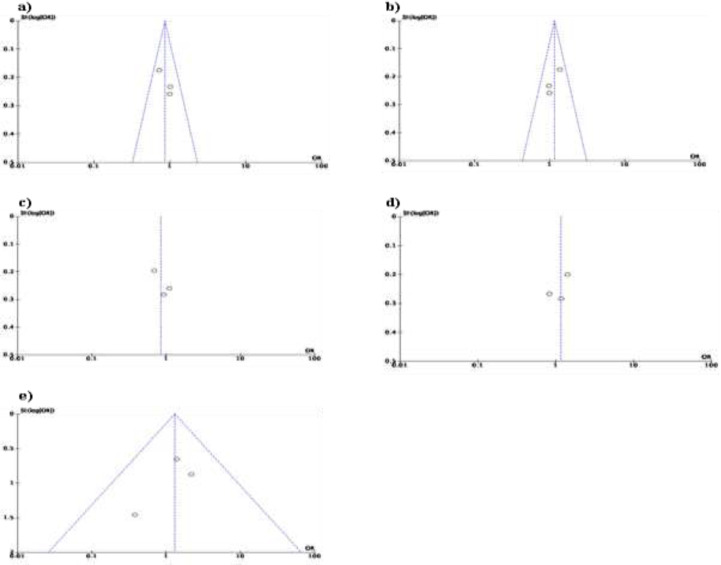
Funnel plot of association between RAGE -429T/C gene polymorphisms and Ulcerative Colitis. a) T vs C; b) C vs T; c) TT vs TC+CC; d) TC vs TT+CC; e) CC vs TT+TC

**Figure 6:**
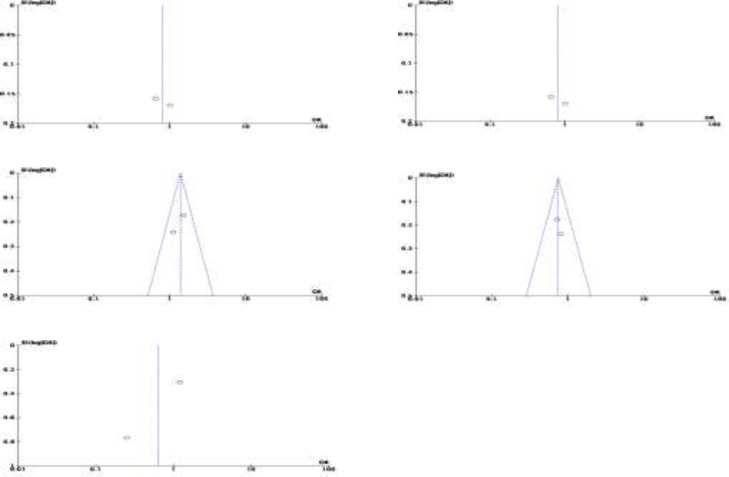
Funnel plot of association between RAGE -374T/A gene polymorphisms and Crohn's Disease. a) T vs A; b) A vs T; c) TT vs TA+AA; d) TA vs TT+AA; e) AA vs TT+TA

**Figure 7:**
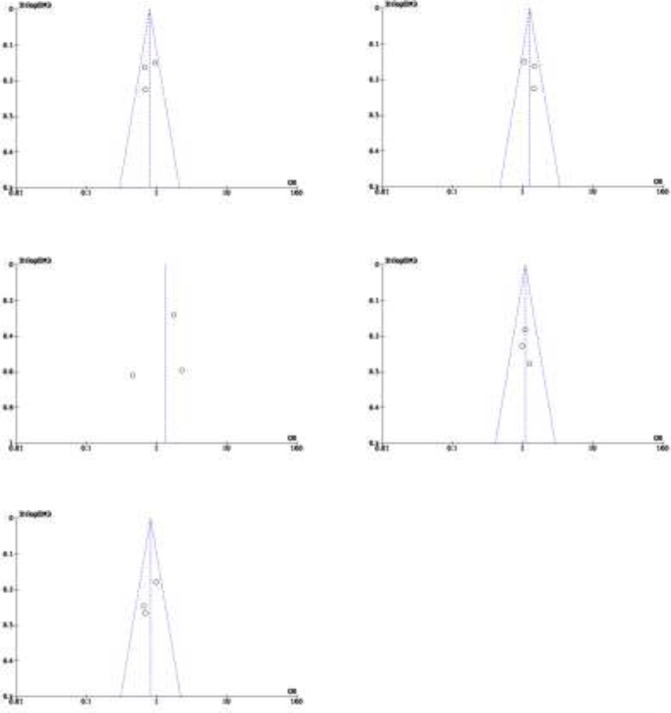
Funnel plot of association between RAGE -374T/A gene polymorphisms and Ulcerative Colitis. a) T vs A; b) A vs T; c) TT vs TA+AA; d) TA vs TT+AA; e) AA vs TT+TA

**Figure 8:**
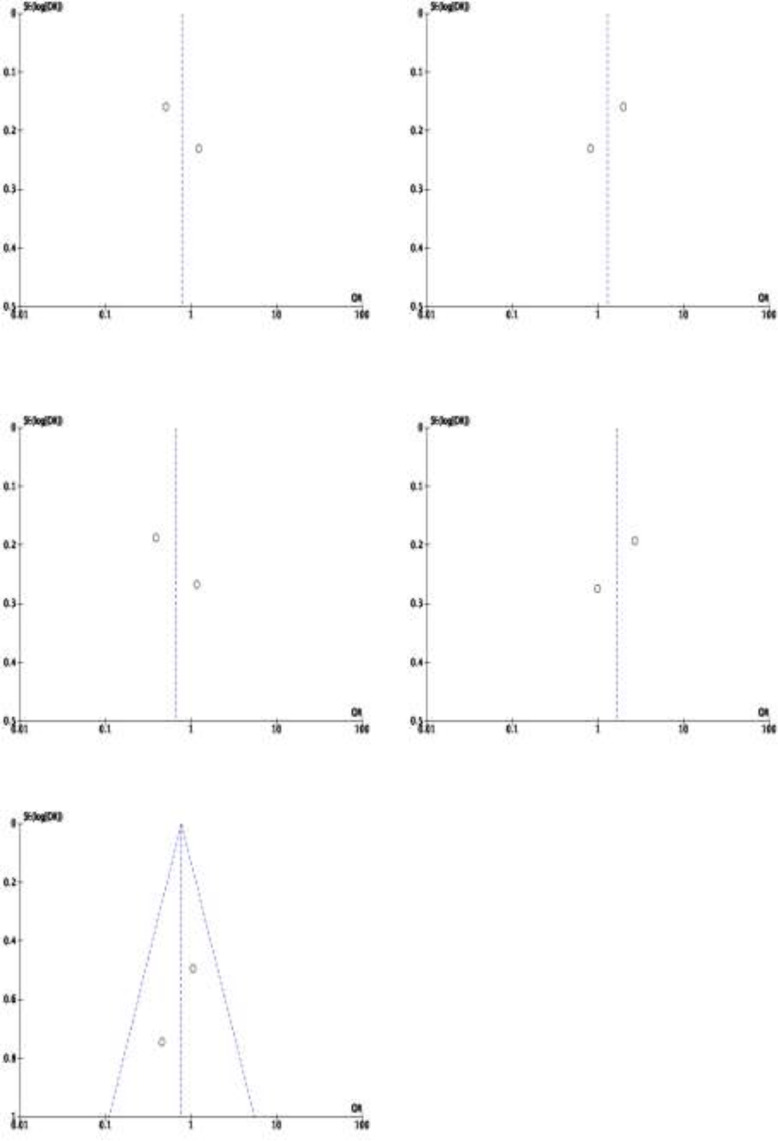
Funnel plot of association between RAGE –G82S gene polymorphisms and Ulcerative Colitis. a) G vs S; b) S vs G; c) GG vs GS+SS; d) GS vs GG+SS; e) SS vs GG+GS

**Bias of Publication: **Additionally, we used funnel plot to assess the possible publication bias for each study included. Publication bias was clearly detected using funnel plots. In contrast, a symmetrical funnel plot demonstrated no publishing bias. The presence of an asymmetric distributed funnel plot shape suggests that publication bias was present. Any genetic model of the researched RAGE SNPs included an assessment of publication bias. The assessment of publication bias is shown in figure 4-8. This result indicates that no potential publication bias shown by symmetrical funnel plots in every genetic marker.

**Table 8:** The summary of the association between RAGE -374T/A gene polymorphisms and the risk of Inflammatory Bowel Disease

**Type of IBS**	**Allele & genotype**	**NS**	**Model**	**Value**		**Sensitivity (%)**	**Specifity (%)**	**OR**	**95%CI**	**p**_h_	**p**
				**Case (%)**	**Control (%)**						
CD	T vs A	2	Random	80.56%	78.40%	80.56%	21.59%	1.24	0.81 – 1.90	0.06	0.32
	A vs T	2	Random	43.03%	21.59%	43.03%	78.40%	0.81	0.53 – 1.24	0.06	0.32
	TT vs TA + AA	2	Fixed	67.41%	62.91%	67.41%	37.08%	1.37	1.04 – 1.81	0.28	0.02
	TA vs TT + AA	2	Fixed	16.31%	19.76%	16.31%	69.01%	0.75	0.57 – 0.99	0.68	0.04
	AA vs TT + TA	2	Random	16.18%	14.13%	16.18%	93.89%	0.64	0.14 – 2.96	0.05	0.57
UC	T vs A	3	Fixed	69.47%	78.31%	69.47%	21.68%	0.79	0.65 – 0.96	0.27	0.02
	A vs T	3	Fixed	54.92%	21.68%	54.92%	78.31%	1.26	1.04 – 1.53	0.27	0.02
	TT vs TA + AA	3	Fixed	50 %	61.85%	50 %	38.14%	0.82	0.64 – 1.05	0.32	0.11
	TA vs TT + AA	3	Fixed	28.75%	20.98%	28.75%	67.15%	1.09	0.85 – 1.40	0.78	0.49
	AA vs TT + TA	3	Random	15.61%	12.23%	15.61%	94.69%	1.33	0.58 – 3.04	0.10	0.50

Notes : NS = Number of sample, OR = odds ratio, CI = confidence interval, p = p value based on a between-study Z test, ph = p value based on Q test for heterogeneity between studies

**Table 9:** The summary of the association between RAGE –G82S gene polymorphisms and the risk of Ulcerative Colitis

**Type of IBS**	**Allele & genotype**	**NS**	**Model**	**Value**		**Sensitivity (%)**	**Specifity (%)**	**OR**	**95%CI**	**p**_h_	**p**
				**Case (%)**	**Control (%)**						
UC	G vs S	2	Random	75.29%	80.71%	75.29%	19.28%	0.77	0.32 – 1.85	0.001	0.57
	S vs G	2	Random	34.64%	19.28%	34.64%	80.71%	1.29	0.54 – 3.09	0.001	0.57
	GG vs GS + SS	2	Random	53.84%	66.39%	53.84%	33.60%	0.66	0.22 – 1.94	0.0007	0.45
	GS vs GG + SS	2	Random	28.48%	17.74%	28.48%	71.34%	1.66	0.64 – 4.42	0.003	0.31
	SS vs GG + GS	2	Fixed	6.58%	12.85%	6.58%	95.04%	0.77	0.36 – 1.67	0.35	0.51

Notes: NS = Number of sample, OR = odds ratio, CI = confidence interval, p = p value based on a between-study Z test, ph = p value based on Q test for heterogeneity between studies

## Discussion

Poor RAGE's function in the pathogenesis of IBD is now under study. IBD is brought on by a complex combination of genetic, immune, and environmental factors. Advanced glycation end products (AGE) are one of the ligands with high binding affinity for the trans-membrane receptor known as RAGE (15-18). RAGE-ligand binding is altered in a number of inflammatory conditions, including cancer. Following RAGE activation, a number of inflammatory pathways are initiated, including nuclear factor kappa B (NF-κB), mitogen-activated protein kinase (MAPK), and Janus activated kinase/signal transducers and activators of transcription (JAK/STAT) (19). RAGE blockage has been proven in prior research to diminish the inflow of inflammatory cells and suppress the release of cytokines (18). 

The RAGE gene, which may be found on chromosome 6p21.3, encodes for RAGE expression. It has been demonstrated that the promotor region's SNPs -374A/T and -429T/C enhance protein synthesis by around thrice and twice, respectively. On the other side, the SNP at the G82S occur in exon 3 of RAGE (20). The relationship between RAGE polymorphisms and IBD has been identified, but the result is still inconclusive. Therefore, we make an effort to weigh the most functional RAGE polymorphisms (-374A/T, -429T/C, and G8S2S). There was no correlation between CD and UC and the G82S polymorphism that showed in table 9. It can be caused due to minimal data. A study by Däbritz et al. also showed no relation between G82S polymorphism and CD (12). This can be caused due to small sample size. Therefore, research with considerably bigger samples is necessary to determine if the G82S polymorphism affects IBD risk in any way (10). Additionally, we discovered no connection between -429T/C and the risk of both CD and UC. According to Wang et al.’s study, there was no correlation between the -429T/C polymorphism and UC, and subgroup analysis insignificantly did not show any differences in the distribution of -429T/C between UC patients and healthy indviduals (21). Besides, *in vitro* study showed that RAGE gene polymorphism in -429T/C increased its expression, around twofold. The expression of RAGE is not only affected by the polymorphism. Concomitant factors such as environment and comorbid could alter the RAGE expression and increase RAGE level in IBD patient. The promotor's genetic polymorphism may be overpowered by this.

 In contrast, we found that some alleles and genotypes were associated with IBD susceptibility. Figure 2 showed that TT genotype of RAGE –374T/A polymorphisms was related to increased CD risk (OR=1.37; 95%CI=1.04 – 1.81; P= 0.02), while in UC (figure 3), A allele of RAGE -374T/A was related to increased risk (OR=1.26; 95%CI=1.04 – 1.53; P= 0.02). In contrast, TA genotype and T allele were served as protective factors, while T allele was related to decreasing CD and UC risk, respectively (OR= 0.75; 95%CI=0.57 – 0.99; P= 0.04 and OR= 0.79; 95%CI=0.65 – 0.96; P= 0.02). Further, research by Ciccocioppo et al. revealed that A allele distribution was higher in UC patient (5). According to reports, RAGE's protein expression and transcriptional activity are both increased by the -374T/A polymorphism. Besides, the binding affinity of the transcription factor was affected by the A allele. Those pathways could increase inflammation through migration of neutrophil in the intestinal epithelium and NF-κB-dependent inflammation factor response (21). Another study showed that polymorphism of -374T/A was a protective factor for CD (12). The increase of RAGE serum could represent either high inflammation or inflammation neutralizer because of its blocking RAGE ligands’ ability (18). Yet, this study’s limitations were: (1) small size of sample, (2) some heterogeneities were found, and (3) the study references were limited. Therefore, the result should be cautiously interpreted. The meta-analyses performed genetic variants, where the TT genotype and A allele of RAGE -374T/A polymorphisms were related with risk of CD and UC, respectively, while the TA genotype and T allele possibly had a protective effect. RAGE –429T/C and RAGE –G82S polymorphisms were not related with an increased risk of IBD. However, in general, the effect sizes were not large, further large-scale, and well-designed studies are needed.
